# A Review of the Role of Bioreactors for iPSCs-Based Tissue-Engineered Articular Cartilage

**DOI:** 10.1007/s13770-023-00573-6

**Published:** 2023-10-20

**Authors:** Alejandro Reina-Mahecha, Martine J. Beers, Hugo C. van der Veen, Inge S. Zuhorn, Theo G. van Kooten, Prashant K. Sharma

**Affiliations:** 1 Department of Biomedical Engineering, University of Groningen, University Medical Center Groningen, FB40, Antonius Deusinglaan -1, 9713AV Groningen, The Netherlands; 2 Department of Orthopedics, University Medical Center Groningen, Groningen, The Netherlands

**Keywords:** Osteoarthritis, Bioreactors, Mechanical stimulation, Tissue engineering, iPSCs

## Abstract

**Background:**

Osteoarthritis (OA) is the most common degenerative joint disease without an ultimate treatment. In a search for novel approaches, tissue engineering (TE) has shown great potential to be an effective way for hyaline cartilage regeneration and repair in advanced stages of OA. Recently, induced pluripotent stem cells (iPSCs) have been appointed to be essential stem cells for degenerative disease treatment because they allow a personalized medicine approach. For clinical translation, bioreactors in combination with iPSCs-engineerd cartilage could match patients needs, serve as platform for large-scale patient specific cartilage production, and be a tool for patient OA modelling and drug screening. Furthermore, to minimize* in vivo* experiments and improve cell differentiation and cartilage extracellular matrix (ECM) deposition, TE combines existing approaches with bioreactors.

**Methods:**

This review summarizes the current understanding of bioreactors and the necessary parameters when they are intended for cartilage TE, focusing on the potential use of iPSCs.

**Results:**

Bioreactors intended for cartilage TE must resemble the joint cavity niche. However, recreating human synovial joints is not trivial because the interactions between various stimuli are not entirely understood.

**Conclusion:**

The use of mechanical and electrical stimulation to differentiate iPSCs, and maintain and test chondrocytes are key stimuli influencing hyaline cartilage homeostasis. Incorporating these stimuli to bioreactors can positively impact cartilage TE approaches and their possibility for posterior translation into the clinics.

## Introduction

Osteoarthritis (OA) is the most common degenerative joint disease [1–3], estimated to affect more than 40 million people across Europe and around 250 million worldwide. Furthermore, it is the fastest-growing cause of disability for elderly and obese patients [4]. OA is characterized by gradual loss and destruction of articular cartilage, variable degrees of synovial inflammation, hypertrophy of the joint capsule, and degeneration of ligaments and menisci [1]. Physical therapy combined with analgesics like paracetamol or nonsteroidal anti-inflammatory drugs are prescribed to alleviate pain and reduce stiffness caused by OA [5]. Oral administration or intra-articular injections of different components present in the cartilage's extracellular matrix (ECM), such as glucosamine, hyaluronic acid, and chondroitin sulfate, are also used to alleviate OA symptoms. Although it is a straightforward procedure to inject therapeutics into joints, intra-articular therapies are compromised by the efficiency and the time interval in which the therapeutical material leaves the synovial cavity [6]. Due to molecule diffusion and the lymphatic system, sustained therapeutic concentrations of drugs are very difficult to maintain in situ. Thus, none of the treatments mentioned above can effectively alleviate the symptoms in the long term nor stop the disease's progression [6, 7].

Ideally, any procedure to repair a cartilage defect should improve the defect by generating a tissue with biomechanical properties similar to the native hyaline cartilage [8]. Multiple surgical techniques are currently being applied to treat focal chondral and osteochondral lesions to prevent OA development. Cartilage debridement, subchondral bone drilling, and osteochondral autologous transfer are a few example procedures showing a regenerative effect with an essential indication of progress [9–13]. However, these techniques often result in fibrocartilage formation or the generation of new morbidity sites and other tissue degenerative changes due to multiple interventions [11]. Among all therapeutic approaches, tissue engineering (TE) has shown great potential as a new therapeutic approach for hyaline cartilage repair in OA. TE aims to create functional constructs that can be used as implants to restore, maintain, or improve damaged articular cartilage [14]. Autologous chondrocyte implantation (ACI) and matrix-induced ACI (MACI) [9] are two techniques that use autologous chondrocytes (Primary cell type of articular cartilage) for implantation in two-step procedures. ACI and MACI are techniques that lack applicability to significant defects. Furthermore, long-term clinical data are scrutinized concerning the quality and durability of the resulting hyaline-like cartilage using these techniques [8, 9].

Looking for more effective approaches, the use of stem cells in TE is exponentially growing. These unspecialized cells can proliferate and differentiate into functional cells of any phenotype [15]. For OA treatment, stem cells are differentiated into chondrocytes. In this case, multiple stem cell types can be considered: mesenchymal stem cells (MSCs), embryonic stem cells (ESCs), and induced pluripotent stem cells (iPSCs) [13].The MSCs’ acquisition procedure is relatively simple, and there are some promising results for articular cartilage repair using this cell type [16]. However, it is questionable whether MSCs are suitable for cartilage TE due to cell ossification and fibrocartilage formation. Instead, both ESCs and iPSCs are pluripotent stem cells, indicating that many cell types in the human body can be derived from them theoretically. ESCs are obtained from the inner cell mass of a blastocyst. However, multiple ethical restrictions are an obstacle to using these stem cells. Therefore, an increased interest exists in the use of iPSCs for TE. IPSCs can be formed from nucleated somatic cells, which are significantly easier to harvest and do not give rise to ethical complications. The harvested somatic cells are reprogrammed into a pluripotent stem cell state [17]. The formation of iPSCs out of somatic cells has been performed using different somatic cell types. The first iPSCs were produced from skin fibroblasts in 2007, and these were transformed into pluripotent stem cells using KLF4, OCT3/4, SOX2, c-MYC or SOX2, OCT3/4, NANOG, and LIN28 genes. The formed iPSCs could differentiate into all three germ layers* in vitro* and* in vivo*, expressing similar gene markers as seen in ESCs [18]. Later, it became clear that iPSCs can also be made from other somatic cells, including white blood cells, cells in the urine, and keratinocytes. These cell sources require non or minimally invasive techniques for their isolation, making them ideal for multiple TE applications [19].

**Fig. 1:**
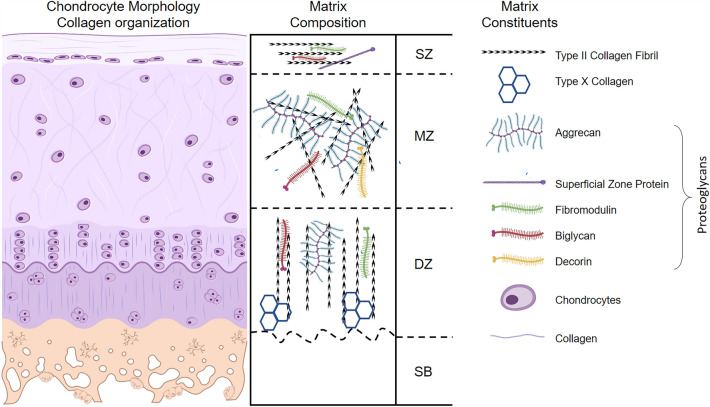
Cartilage schematic representation with matrix composition and matrix constituents. SZ: Superficial Zone, MZ: Middle Zone, DZ: Deep Zone, and SB: Subchondral Bone. Figure created partially with Biorender.com

For OA research, pluripotent stem cell (PSCs) differentiation into chondrocytes has been performed using either cytokines or cell stimulation [19]. For the latter approach, bioreactors are commonly used to stimulate mechanosensitive pathways for stem cell differentiation. A bioreactor is a device that primarily aims to mimic the* in vivo* environment of chondrocytes and articular cartilage [20, 21]. Therefore, it is essential to understand the nature of this complex tissue. In its structure, articular cartilage is an organized tri-zonal tissue composed of glycoproteins made of sulfated glycosaminoglycans (GAGs) connected to a backbone of HA, collagen (mainly type II), and water in its extracellular matrix (ECM) and chondrocytes. In each zone of this tissue (Fig. 1), these components' concentration, orientation, and interaction vary to provide a viscoelastic, load-bearing, low-friction behavior and its necessary strength to distribute and withstand daily variable mechanical forces [22, 23].

Bioreactors often control pH, temperature, nutrient supply, oxygen levels, and waste removal and use mechanical, electrical, or magnetic stimulation [24, 25]. These factors help the tissue be studied under physiological and pathophysiological conditions. Ideally, iPSCs intended for engineering articular cartilage under exposure to bioreactors should differentiate towards chondrocyte cells capable of synthesizing and depositing collagen type II to guarantee tissue stability. Furthermore, produce proteoglycans, e.g., GAGs which, due to their negative charge, can interact with the polar water molecules to control the interstitial flow through the ECM pores, giving its viscoelastic load-bearing capacity to withstand the mechanical forces. Ultimately, after bioreactor-controlled exposure, the cartilage construct should exhibit the tri-zonal organization to form a functional interface for native tissue integration [23].

For* in vitro* testing of tissue-engineered hyaline cartilage, a bioreactor must be designed to resemble the* in vivo* conditions of the (diseased) joint cavities [26]. Articular tissues, especially articular cartilage, are simultaneously exposed to metabolic, mechanical, and biochemical stimuli, including ion exchange. However, recreating human joints* in vitro* is not trivial because the interactions between various stimuli have yet to be entirely understood. Furthermore, they are difficult to be mimicked in a bioreactor. Thus, for optimal articular cartilage reconstruction, some assumptions must be considered for complex parameters, such as mechanical and electrical stimulation. This review discusses the necessary parameters for a bioreactor intended for cartilage TE, focusing on the potential use of iPSCs. Furthermore, it focuses on using mechanical and electrical stimulation to differentiate, maintain, and test chondrocytes and tissue-engineered articular cartilage under* in vitro* conditions.

## Methods

A PubMed search was performed under the search terms (Osteoarthritis AND Treatment) OR (Bioreactor AND Cartilage) OR (Bioreactor AND Tissue Engineering) OR (Cartilage AND iPSCs) and a combination of those terms, with results limited to the English language and published in the last ten years. Other articles used in this review were taken as a result of cross-referencing. A complementary PubMed search under (Bioreactor AND Cartilage AND Tissue Engineering) was performed, resulting in 287 articles. Articles that did not address hyaline cartilage or osteoarthritis were not considered. For this review, the article choice was based on the journal impact factors, translatability to newer and novel technologies, the potential impact of the study, and opinions from experts in cartilage tissue engineering. Thus, in total, 85 articles were included in this systematic review.

### Bioreactors for* in vitro* tissue-engineered articular cartilage optimized development

Tissue-engineered cartilage constructs can be formed from multiple stem cell types and numerous techniques [19]. A standard medical implant testing method is* in vitro* experiments, followed by* in vivo* animal testing before clinical trials.* In vitro,* research investigates the applicability, toxicity, and biocompatibility of tissue-engineered implants to reduce possible complications of* in vivo* applications. This screening is necessary because 90% of biomedical research studies fail to enter routine clinical use even after long testing periods (~ 20 years) [27].

The use of monolayer cell cultures often is the starting point for investigating the potential of TE strategies. However, these cultures do not resemble the dynamic complex environment of the tissue* in vivo*. Therefore, 3D cell culture models have recently been used to analyze microenvironmental cues that can regulate biochemical responses for chondrocyte gene expression and cell differentiation [28].

For assessing and testing the interplay between tissue, biological, chemical, and mechanical stimuli, a bioreactor is a desirable device to mimic multiple characteristics of the* in vivo* milieu [29]. In this way, it can be found whether the obtained cartilage tissue has the potential to be implanted or not [21]. E.g., When chondrocytes cannot withstand the bioreactor's environment, they usually start to express genes and cell markers that cause differentiation to other cell phenotypes or cause cell death. If this happens, the cartilage-engineered constructs have a low chance of surviving* in vivo* and will not make it into further stages to reach clinical trials [30].

Using a bioreactor for screening tissue-engineered cartilage constructs can accelerate the translational process for more clinically relevant research [31]. However, a bioreactor design is not trivial, and its validation can be challenging due to multiple parameters that can be considered (Table 1) to resemble a big part of the* in vivo* environment for cartilage differentiation, regeneration, and maintenance.

**Table 1:** Summary of representative stimulus to consider for the design of a bioreactor for tissue-engineered articular cartilage

Type of stimulus	Range	Continuous/intermittent	Results	Synergism/antagonism	Ref
Flow rate	0.01–4 ml/min	Continuous	The optimal values for nutrient distribution, oxygen, and waste removal would range from 0.2 to 1 ml/min. The seeded scaffold exhibited higher DNA content and enhanced GAG deposition	Waste removal	[32]
pH	6.6–7.4	Continuous	Buffering stimulated ECM deposition and enhanced cell proliferation	Glucose and O2 concentrations	[33–35]
Waste removal		Intermittent	In static culture, significant diffusion resistance exists. This prevents efficient transport of waste out of the cartilage construct. Dynamic flow promotes waste removal and stimulates chondrocyte proliferation	Flow rate	[1, 32]
O_2_ tension	~ 5%	Continuous	Low oxygen tension conditions promote collagen type II and GAG deposition and promote chondrogenic markers expression	CO2 tension	[36–38]
Growth factors	5–10 ng/ml	Continuous	GDF-5 increases GAG biosynthetic activity, BMPs promote chondrogenic aggregates and chondrocyte differentiation, and TGF-β allows prechondrogenic condensation and maintains cartilage integrity		[39–41]
Shear stress	1–3%	Intermittent 1 Hz -1 h/day	MMP and ADAMTs downregulation. Upregulates aggrecan expression	Compressive stress	[26, 42]
Compressive stress	0–5 N	Intermittent 0.01-1 Hz—1 h/day	MMP and ADAMTs downregulation. Upregulates aggrecan expression	Shear stress	[26, 43]
Hydrostatic pressure	7–10 MPa	Continuous	Cartilage ECM synthesis, faster ECM accumulation, prolonged cell survival, increment in chondrogenic gene expression	Alter the O_2_ tension	[37, 44–46]
Electrical stimulus	5 V/cm	Intermittent (8 ms pulses at 5 Hz)	Creates ATP oscillations, leading to prechondrogenic condensation, enhances expression of chondrogenic markers like Sox9, modulates cAMP levels that promote chondrogenic proliferation and anti-inflammatory pathways	Alter the pH due to electrolysis	[47, 48]

In 1959, Russel and Burch proposed improving animal welfare through an initiative called the 3Rs: "replacement, reduction, and refinement." The 3Rs aimed to reduce the number of animal studies by using other testing methods [49]. Thus, another significant advantage of bioreactors’* in vitro* screening of tissue-engineered constructs is reducing the number of animals required for* in vivo* trials. If these articular cartilage constructs fail or do not respond positively to the bioreactor stimuli, the constructs need to be neglected for animal testing [29]. In humans, the articular cartilage is a mechanically active tissue that must withstand approximately three times the individual’s body weight [50].

Without bioreactors, the constructed tissues should be tested in animals, which in this case, represent the tissues' *in vivo* environment. These tests are mainly performed on small animals, like mice, rats, and rabbits [29]. By doing this, the tissue response related to the biological factors can be investigated. However, these small animals cannot expose the tissue to the same forces and other stimuli as humans [20]. Therefore, large animal studies are preferred for* in vivo* translations.

According to Peroglio et al. [29], a bioreactor, including a control unit and software, can be built for the same amount of money as performing a study with ten large animals. They calculated that testing on a large animal model costs around nine thousand dollars per animal. Thus, ten large animals can be used with a budget of about 100,000 dollars. However, for the same money, a sophisticated bioreactor can be built [29]. Furthermore, it is a system that can be used multiple times and has a broader range of applications. In this manner, bioreactors can considerably reduce the amount of animal research and consequently reduce research costs [30], specifically for articular cartilage TE on OA research.

Another advantage of reducing the number of animals for research is that it saves experimental time. Before performing an animal experiment, a researcher must not conflict with any animal ethics related to the experiments. Therefore, an application to the respective institution´s animal ethical committee must be sent, including details about the project and the intended animal use [51]. The time between the application and the start of the experiment can include a long waiting time, which could be saved using bioreactors for* in vitro* testing [52]. In that way, it is possible to test faster multiple cartilage constructs created out of different cell lines and techniques compared to animal studies. Therefore, by using a bioreactor, tissue-engineered cartilage development can be accelerated [31].

**Fig. 2:**
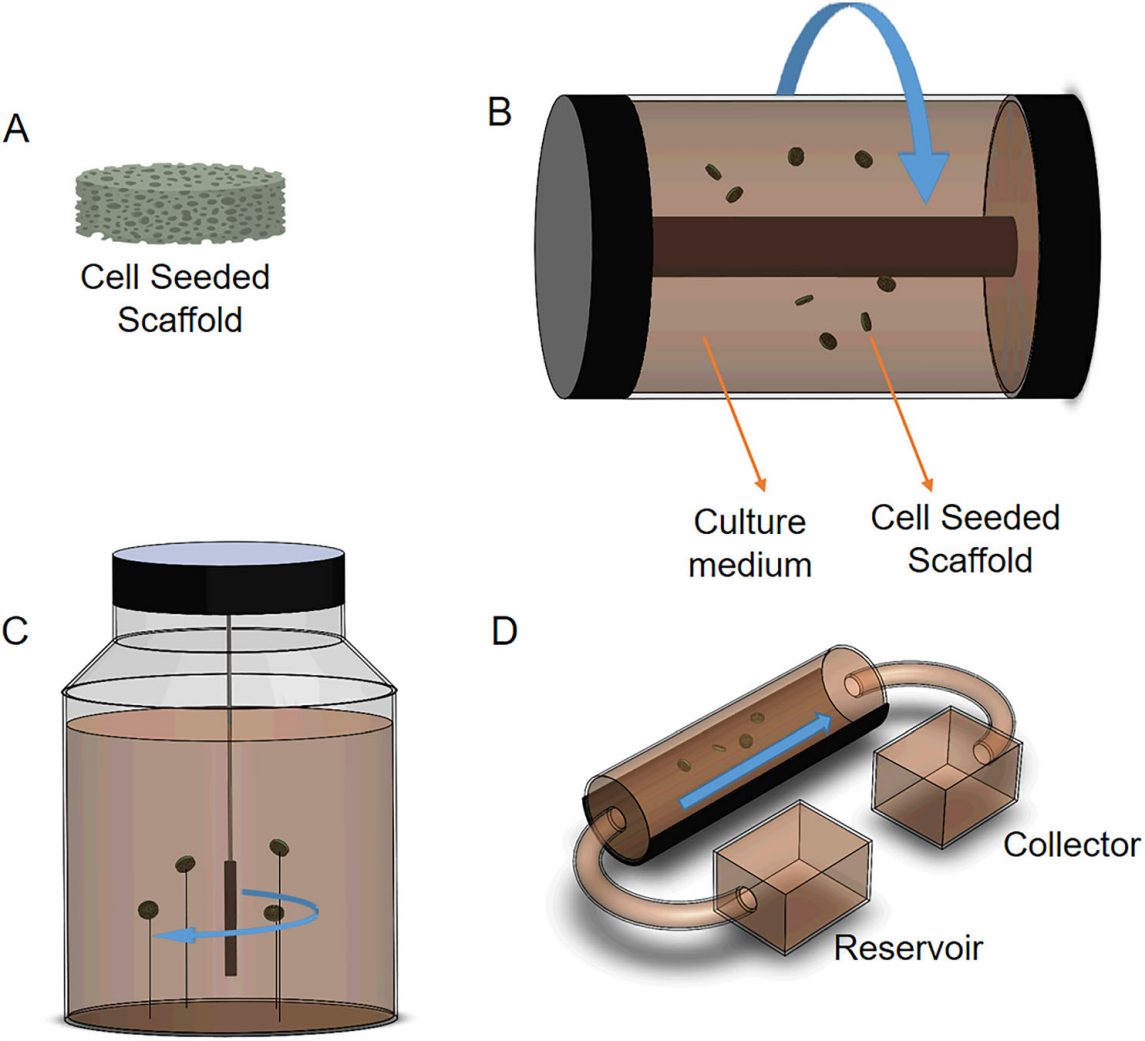
Schematic representation of **A** cell-seeded scaffold, **B** Rotating wall vessel bioreactor, **C** Spinner flask bioreactor, and **D** Flow perfusion bioreactor

Three basic bioreactors have been commonly used to engineer multiple tissues in the lab, including hyaline cartilage. A schematic overview of those bioreactors can be found in Fig. 2. In this review, it is emphasized the use of iPSCs cell-seeded scaffolds in combination with bioreactors. However, other 3D systems, such as embryoid bodies [53] or iPSCs-derived cartilage organoids [54], are encouraged to be used for* in vitro* cartilage fabrication and OA research.

Scaffolds play a significant role in cartilage TE by creating an ideal cellular environment to enhance tissue integration. Essential features, including scaffold architecture, material biocompatibility, biodegradability, and chemical and mechanical properties of the scaffold, can be achieved by multiple manufacturing methods. Essential information and description of these requirements for cartilage TE can be found elsewhere [55].

The rotating wall vessel (RWV) (Fig. 2B) bioreactor consists of two concentric cylinders with space in between, suitable for the cell-seeded porous scaffold (Fig. 2A) location. The wall rotation induces a laminar fluid flow, facilitating oxygen diffusion and nutrient supply through the scaffold [56]. Studies have demonstrated higher glycosaminoglycans (GAG) and collagen II expressions using RWV on cartilage constructs [57, 58].

The spinner flask is a simple bioreactor that uses a magnetic stir bar to gently agitate the medium where needle-extended cell-seeded scaffolds are cultured (Fig. 2C). Medium agitation helps nutrient and oxygen supply throughout the cell-seeded scaffolds (Fig. 2C), allowing an increment of cell proliferation, GAG content, and collagen II compared to static cultures for cartilage TE [59]. In both RWV and spinner flask bioreactors, to reduce cell waste, 50% of the medium must be replaced every other day during the culture time [25]. Therefore, the flow perfusion bioreactor (Fig. 2D) is one alternative for cartilage TE constructs. This system uses a controlled pump that allows the medium to be perfused through the cell-seeded scaffold, favoring nutrient supply and waste removal [60]. Flow rates between 0.2 and 1 mL/min have demonstrated good cell viability and ideal cell waste removal. Furthermore, This bioreactor has shown a significant increment in DNA and GAG content on Poly-L-lactic acid scaffolds for cartilage formation [32].

Furthermore, bioreactors can also be used for OA drug research. For this application, it is essential to identify drug candidates for potential OA treatments and harmful compounds that can further promote the progression of the pathology. Currently, for drug testing, cell-loaded scaffolds or cell organoids are used in combination with flow perfusion bioreactors, where cell responses to specific drugs can be monitored. E.g., Nichols et al. developed a 3D-printed bioreactor that allows the multidirectional flow of medium containing potential screening drugs through the cell-loaded scaffold [61]. This bioreactor uses a complex microfluidic system that optimizes the drug-medium supply, resembling* in vivo* conditions. Furthermore, it has optical access for drug response monitoring, which can be used for comparison prior to or in parallel with animal studies to increase the profile safety of the screening candidate drugs [14, 61].

### Biochemical and Physical stimuli necessary for tissue-engineered articular cartilage

The increased interest in using iPSCs for TE approaches, including cartilage regeneration, brings to play multiple biochemical and physiological stimuli that can promote chondrogenesis in the cell's pluripotent state. In the embryo, the formation of cartilaginous anlage happens from the condensation of mesenchymal progenitors. This condensation requires hypoxic environments, making oxygen concentration an essential parameter in a bioreactor environment. Studies using hypoxic conditions (O_2_ 5%) have demonstrated enhanced chondrogenic differentiation [36, 38, 62].

Other conditions, like temperature and pH level, can be easily incorporated into bioreactors simultaneously. Electrical-resistor heat dissipation systems with feedback control are typically used to keep TE constructs under constant temperature. The human body is a system that maintains an average temperature of approximately 37 °C. However, in healthy and osteoarthritic conditions, articulations like the knee can experience a temperature variation [63]. Therefore, TE applications for this particular case should target* in vitro* temperature culture conditions using bioreactors.

Previous research on articular pH showed that synovial fluid (SF) is the major interactor of the articular cavities. The SF, which keeps an average pH of 7.4, delivers by diffusion nutrients and oxygen necessary to the correct function of articular cartilage [33–35]. For iPSCs culturing and differentiation towards chondrocytes, a defined chondrogenic medium (CDM) can supply certain nutrients and growth factors frequently used to stimulate the growth and preservation of tissue [39–41]. An advantage of using commercially available basal mediums is maintaining physiological pH levels under 5–10% CO_2_ conditions [60]. Table 1 summarizes the most important biochemical and physical stimulus for* in vitro* cartilage regeneration.

### Mechanical stimulation for tissue-engineered articular cartilage

Another advantage of bioreactors for* in vitro* testing is developing an excellent rehabilitation program after cartilage implantation. By studying the effect of particular stimuli in the bioreactor, conclusions can be made about which stimuli positively or negatively impact specific diseases, as is OA [21].

Mechanical loading of the joints is an essential factor to study. Besides the growth factors and cytokines, the chondrocytes respond to loading by appropriately synthesizing the required molecules, such as type II collagen and proteoglycans [64]. The chondrocyte's mechanical loading mitigates joint destruction by downregulating matrix metalloproteinases (MMPs) and a disintegrin and metalloproteinase with thrombospondin motifs (ADAMTS) [65]. Besides the downregulation of MMPs and ADAMTS, mechanical stimulation increases aggrecan expression, the most abundant glycoprotein in the ECM [66]. The balance in ECM production in healthy articular cartilage tissue depends on a stable phenotype of the chondrocytes. Cartilage under cyclic (0.002–0.01 Hz) compressive strain amplitudes of 10% [26, 43] (corresponding to normal stresses of 0.5 MPa) and intermittent hydrostatic pressures of 1–10 MPa enhanced protein and proteoglycan biosynthesis [67]. Cartilage under constant 10% compressive strain and cyclic shear strain of 1–3% (corresponding to 10–20 kPa shear stress) at a frequency of 0.01 to 1 Hz has doubled protein synthesis, and 25% increased proteoglycan synthesis [26, 42]. A shear strain of around 1% promotes regenerative pathways for cartilage regeneration by promoting chondrocytes-ECM homeostasis [68]. In contrast, increased friction at the cartilage surface may cause much higher shear strains leading to chondrocyte catabolic activity.

Chondrocytes are phenotypically unstable. Therefore, a lack of mechanical loading can result in differentiation to other cell phenotypes, which induces different ECM proteins' production, leading to low-grade tissue mechanical properties. This phenotypic instability is a significant problem in OA development and a characteristic considered in TE. Appropriate mechanical stress on chondrocytes can give the cells valid signals for producing the components necessary for the load-bearing of the ECM [65].

Although a balance between too much and too little mechanical loading is relevant for creating TE solutions combined with bioreactors, excessive compressive and shear load over the hyaline tissue can activate biological pathways that promote articular cartilage degeneration. However, cartilage degeneration accelerates without mechanical stimulation due to softening and thinning of the articular cartilage and decreased glycosaminoglycan content in the joint [69].

**Fig. 3:**
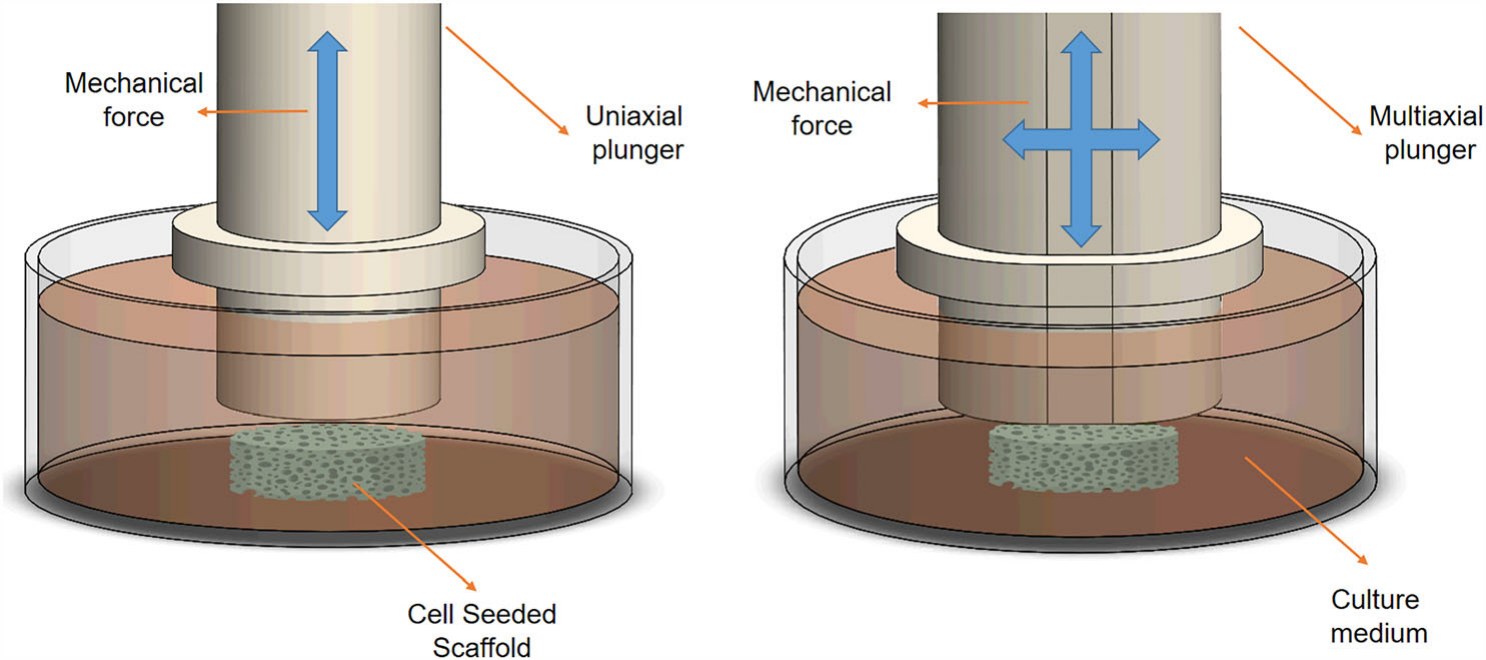
Schematic representation of mechanical stimulation for tissue engineering constructs

For mechanical stimuli (Fig. 3), the bioreactor should mimic the forces generated by the joints' multiaxial movements, simulating the kinematics and mechanical loading* in vivo* [29]. A continuously changing stress is applied to the articular cartilage for lower limb articulations. Thus, the amount of stress directly depends on the person's weight and other gait factors. People at risk of developing OA are most likely people suffering from obesity. Therefore, exposing the* in vitro* cartilage to high mechanical loads can be beneficial in investigating whether the iPSCs-engineered cartilage can withstand the ultimate mechanical load and identify which biological routes can be addressed for further research [70].

Both static and dynamic compression have shown a chondrogenic gene expression increment compared to non-stimulated constructs. However, dynamic compression has the advantage of increasing chondrogenic differentiation markers in stem cells, as it resembles the function of articular cartilage during motion [71–73]. In dynamic compression, a uniaxial or multiaxial mechanical stimulus can be applied. Making the correct assumptions for these stimuli to mimic human articulations is challenging. Therefore, analyzing these parameters with models and computational tools is helpful instead of using a trial-and-error method [24].

An advantage of using the multiaxial is that it can better replicate the physiological conditions of cartilage, allowing a more accurate simulation of its natural growth and function. The multiaxial stimulus exhibits a higher expression of chondrogenic marker genes [26]. Furthermore, it also improves tissue functionality by promoting a broader range of mechanotransduction pathways, enhancing cell differentiation, ECM deposition, and tissue maturation compared to the uniaxial stimulus [74]. The enhancement of ECM matrix deposition is translated into an increase in collagen type II and proteoglycan (GAGs) deposition; both components can enhance the mechanical properties of the engineered constructs and facilitate tissue integration in case of implantation [75].

Mechanical forces play a significant role in forming joint cavities in embryonic skeletal development. Absence or restricted fetal movement and mechanical force exertion can result in multiple syndromes such as dystrophy and muscle atrophy [76]. Incorporating iPSCs into articular cartilage TE combined with bioreactors is a potential research field to identify mechanobiology routes expressed by the cells in the pluripotent state [23, 77].

### Hydrostatic pressure for tissue-engineered articular cartilage

Hydrostatic pressure (HP) is a physical stimulus present in daily motion articulation activities due to a relationship between the proteoglycan aggregates and interstitial fluid. This relationship provides cartilage compressive resistance through negative electrostatic forces. The proteoglycan fixed charge determines the ECM ion composition. As this ion concentration is higher than in the synovial fluid, the difference results in EMC fluid intake creating HP [78]. In OA patients, the articulations are affected by changes in the quantity and quality of the synovial fluid. A mismatch of the fluid with daily activities can affect the native HP of the joints and, ultimately, the tissue homeostasis. Using controlled HP generated by bioreactors has increased* in vitro* cartilage ECM synthesis on engineered constructs. The presence of a 7–10 MPa HP showed faster ECM accumulation, prolonged cell survival, and increased chondrogenic gene expression[37, 44–46]. Furthermore, HP has been proven to increment the Collagen II and glycosaminoglycan (GAG) content on cartilage TE approaches [65]. Studies using HP bioreactors on PSCs lines have to consider that this physical stimulus can affect the oxygen solubility, thus, affecting the oxygen tension and ultimately forming cartilaginous anlage under hypoxic conditions [37, 46].

### Electrical stimulation for tissue-engineered articular cartilage

As hyaline Cartilage is an avascular tissue, the synovial fluid is responsible for chondrocyte nutrition and maintenance. The cartilage's ECM has abundantly fixed ionized macromolecules that interact with the synovial fluid to create diffusion and strain electric potentials, resulting in essential signals for tissue homeostasis [47]. In OA, unbalanced mechanical forces are present in the articulation due to cartilage loss and synovium inflammation, and thus, the force mismatch harms the SF interaction over the articular cavity. The ECM disruption present in this pathology together with inflammatory proceses create an ionic imbalance of the cartilage environment, with the presumption of electrical field disruption affecting tissue maintenance and changes in the chondrogenic phenotype of the cells [47, 79].

**Fig. 4:**
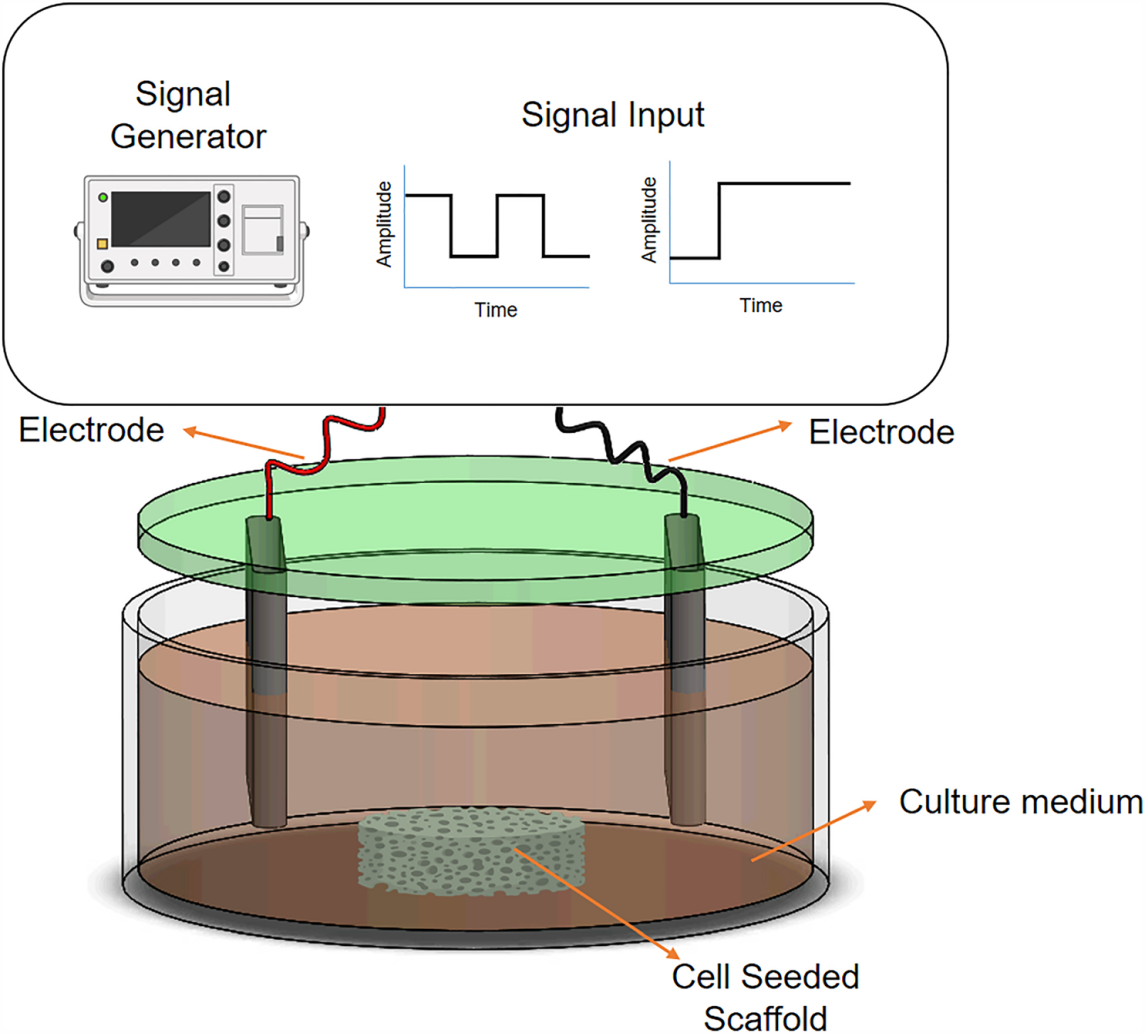
Schematic representation of electrical stimulation with an interval and a continuous step signal input for tissue engineering constructs. Figure partially created with Biorender.com

Regenerative pathways for cartilage regeneration on iPSCs engineered constructs can be stimulated by endogenous electrical stimulation (ES, Fig. 4), which induces* in vitro* chondrogenesis and* in vivo* cartilage repair, which is demontrated by the results described below.

After incorporating ES on stem cells, this stimulus generates ATP oscillations driven by Ca^2+^ oscillations. These ATP oscillations are crucial for prechondrogenic condensation and posterior expression of chondrogenic markers such as collagen II, aggrecan, and Sox9 [79, 80]. Furthermore, when applied in pulses, ES can modulate the cyclic adenosine monophosphate (cAMP) levels by activating adenosine receptors that promote chondrocyte proliferation and anti-inflammatory pathways [48].

Kwon et al. demonstrated that ES also drives ATP oscillations by cAMP modulation, leading to chondrogenic differentiation in the absence of exogenous growth factors [79]. To introduce ES* in vitro,* the bioreactor needs to mimic the electrical environment of the articulations. Although this stimulus is present permanently in the cartilage tissue, on* in vitro* cartilage regeneration or development using iPSCs, the stimulation must be controlled by frequency, duration, amplitude, and type of stimulation (Table 1). Additionally, the bioreactor's components to deliver the ES need to be specifically designed to avoid electrolysis, altering the pH of the medium and modifying cell differentiation. The design must consider the proper electrode placement to ensure the minimal contact with the iPSCs to enhance cell viability. Ideally, the electrodes must be made of carbon as it is widely known that this material hardly corrodes, minimizing the chances of electrolysis. Furthermore, any ES bioreactor intended for iPSCs applications must contain a proper grounding and an electrical current control system to prevent and rectify the system in case of electrolysis.

## Discussion

Bioreactors can be one of the most efficient and reliable methods for testing* in vitro* articular cartilage-engineered constructs. Unlike static 2D or 3D cultures, these devices can resemble some dynamic stimuli and conditions present in articulations for the optimal development of articular cartilage. After controlling and optimizing specific electric and mechanical stimuli, TE cartilage constructs, including the correct stem cell type, can increase the expression of chondrogenic genes and markers as present in healthy tissue. In this way, tissue constructs can increase the ECM deposition of essential cartilage components like collage type II and proteoglycans. The presence of these components in articular cartilage constructs enhances the mechanical properties and can facilitate tissue integration in case of* in vivo* implantation. However, the optimization of these or other stimuli has multiple limitations.

Pluripotent stem cells, like multiple other cell types in the human body, are mechanosensitive, meaning that mechanical forces regulate and remodel the tissue ECM. At the cellular level, cells sense, exert, respond, and decipher physical forces; meanwhile, at the molecular level, mechanosensitivity elucidates the recruitment and interconnection of molecular players to trigger specific biological functions [81]. In the case of basic bioreactors like the RWV, the free-falling state created by the rotation of the walls drastically diminishes the mechanical stimulation to the cartilage tissue constructs, in the long term, inducing cell differentiation into other cell phenotypes different from chondrocytes [82]. In the case of the spinner flask bioreactor, the rotation of the magnetic bar can create turbulent flows that can affect the cell-to-cell or cell-to-scaffold interaction, reducing the amount of ECM deposition. Controlling and adjusting the optimal medium circulation through the flask without disturbing the cartilage tissue constructs can be challenging [83].

For the stimulation of TE cartilage using more complex bioreactors, first, it needs to be considered the complexity of articular cartilage and its environment. As multiple variables and underlying biological processes happen, bioreactors are limited to only deliver some of the most essential and most effective physical stimuli that can be applied to engineered constructs. In OA, it is known that excessive mechanical loading enhances OA through the activation of downstream catabolic pathways that stimulate the production of MMPs and ADAMTS [84]. Thus, finding the relative force that needs to be applied to the* in vitro* cartilage can be a limitation not only to the mechanosensitive nature of the iPSCs but also to identify the mechanical limit of the constructs.

For bioreactors intended for ES, constructs can easily be placed between electrodes, and the desired signal, frequency, and amplitudes can be delivered. However, electrical signals can be found in multiple other tissues in the human body, e.g., the heart, muscles, and neurons. Therefore, this physical stimulation must be optimized for cartilage differentiation, especially for iPSCs and their high sensitivity to physical signals. Furthermore, different from the biological site, integrating ES can be challenging as the culture system's temperature and pH need to be maintained. For the temperature, the fluid acts as a resistor that dissipates heat, producing a relative temperature increment that can disturb the correct function of the cells in the cartilage construct. For the pH, the electrical interaction with the medium produces hydrolysis, liberating ions that can affect the system's pH. As iPSCs are so sensitive to their microenvironment, pH changes can alter their protein folding, enzymatic activity, and gene expression. This leads to undesired cell functionality and the potential risk of iPSCs differentiation towards other cell phenotypes [85].

Other challenges in designing complex bioreactors for delivering multiple stimuli are system sterility, biocompatibility, scalability, and monitoring. Incorporating all these properties would result in significant investments that laboratories must pay. However, current technologies like 3D printing and 3D stereolithography (SLA) are alternatives that can help with significant price reduction. Nowadays, multiple biocompatible resins used for SLA offer exceptional mechanical properties that are preserved even after sterilization, helping reduce bioreactor fabrication costs.

Based on the available literature, we envisage an ideal bioreactor for iPSCs-cartilage TE, a device that, besides standard culture conditions such as controlled temperature (37C), oxygen tension (5% during differentiation), and CO_2_ level (5%), can provide simultaneously mechanical and ES that will be controlled following a specific frequency, intensity, and duration of the stimulus (Table 1). The device could be ideally 3D printed using sterilizable biocompatible resins or polymers for efficient prototyping and cost reduction. Furthermore, it should be able to process multiple samples simultaneously and finally contain a nutrient supply and waste removal to mimic the* in vivo* joint conditions as much as possible.

As perspective, successful incorporation of bioreactors with iPSCs-Cartilage constructs will significantly impact clinical translation for OA treatment. As iPSCs can be derived from a patient's cells in the early stages of OA, these devices can serve as a platform for personalized drug testing and therapeutic interventions to mitigate the progression of the disease. Additionally, bioreactors can provide a controlled environment to differentiate iPSCs-cartilage constructs into functional articular cartilage matching the patient´s specific needs. Furthermore, in advanced OA, they can also facilitate the scalability of cartilage tissue production for implantation, which is beneficial for the clinical translation of OA treatment, where the pathology damages a large amount of cartilage.

## Conclusions

In conclusion, this review highlights the importance of mechanical and electrical stimulation that can substantially impact iPSCs differentiation through bioreactors. These devices have been researched in cartilage tissue engineering with promising results for the* in vitro* evaluation of cartilage formation, regeneration, and potential translation to the clinics. To ensure progress in this field and profiting the differentiation capacity of iPSCs, researchers must continue to be encouraged to use iPSCs in combination with bioreactors to enhance the treatment of OA for the increasing number of patients suffering from this debilitating disease.

## Data Availability

The datasets used and/or analyzed during the present study are available from the corresponding author on reasonable request.
